# Metabolic Effects on Body Components After a Three-Month Physical Intervention in Overweight Medical Staff

**DOI:** 10.7759/cureus.19027

**Published:** 2021-10-25

**Authors:** Song Wen, Shuren Xu, Thiquynhnga Nguyen, Min Gong, Huafang Yan, Ligang Zhou

**Affiliations:** 1 Endocrinology, Shanghai Pudong Hospital, Shanghai, CHN; 2 Physical Examination Center, Shanghai Pudong Hospital, Shanghai, CHN

**Keywords:** body fat, metabolic effects, body component analyzer, physical interventions, obesity

## Abstract

Purpose

This study analyzes the metabolic effects on body components of short-term standard physical training interventions in the staff of a Chinese hospital.

Methods and materials

We analyzed annual medical examinations, including blood sampling, ultrasound examinations, etc., and selected 10 overweight voluntary participants to take part in formal physical training, and a body composition analyzer DBA-550 (Donghuayuan Medical Co., Ltd, Beijing, China) was used to analyze body components' change before physical training interventions and the first month and third month after the physical intervention.

Results

The intervention significantly decreased body mass index (BMI) (p<0.05). Plasma lipids, triglyceride, and waist/hip ratio in females, trunk circumference in males, and limb circumference in females changed significantly (p<0.05). The body composition analysis showed that alterations in lean mass, fat weight, and fat percentage were not significant. Moreover, the segmental skeletal weight stable and segmental edema indices changed significantly but were within the normal range.

Conclusions

Three months of short-term physical intervention effectively lower body weight and fat, but more significant changes in long-term intervention and larger groups can be expected. Besides, the body composition analyzer proved reliable and can modify more individualized treatment plans for overweight and obese individuals.

## Introduction

Modern society has brought about many more lifestyle-related diseases in an unprecedented manner. Among others, obesity is the most common condition. Its incidence increases, especially when there are increased chances of accessing more calories and adapting to insufficient energy expenditure [1]. In 2016, the Non-Communicated Diseases (NCD) Risk Factor Collaboration reported the age-standardized worldwide prevalence of obesity (defined as a body mass index (BMI) ≥30 kg/m^2^) in 2014 in men and women was 10.8% and 15.0%, respectively, and the prevalence of overweight (BMI between 25 and 30 kg/m^2^) was 24.4% and 27.9% [2]. It is estimated to rise to 18% and 21% by 2025 if it continues in the current conditions with no effective control [2]. On the other hand, overweight and obesity in Chinese adults is about 70% [3].

Our previously published review discussed the pathophysiology and detrimental effects of obesity, including type 2 diabetes, cardiovascular diseases, chronic kidney insufficiency, and even cancer [4]. Obesity, incredibly visceral fat accumulation, is more harmful to systemic well-being [5]. However, over or too-fast body weight loss may also be detrimental to body organs such as the liver [6]. Therefore, an ideal body weight loss target is crucial. Previous studies reported that 5% or more of body weight reduction could prevent the risk of cardiovascular complications [7-8]. Fortunately, the current treatment options are overwhelming, including pharmacotherapy like incretin-based therapy, SGLT-2 inhibitors, surgery such as sleeve gastrectomy, etc. [9]. Understanding the indications for each therapeutic option is essential as well. Although these options are powerful in the short duration after treatment, lifestyle scheduling should be more critical over a long time. Current strategies for lifestyle control may vary, the majority include diet and exercise, but the question on which of the two strategies is more suitable and moderate remains unclear, especially considering the differences in gender, age, and job [10]. To our knowledge, data on weight reduction among the staff of hospitals were scarce [11]. Therefore, we implemented this study to explore the effectiveness of short-term physical training interventions in obese populations. In order to quantify and evaluate the interventions, we utilized the body composition analyzer in concert with medical examinations such as blood sampling, ultrasound examinations, etc.

The measurements of human body composition are objective methods for nutritional assessment and are of interest to nutritionists, health professionals, and sports scientists. Body composition measurements can help evaluate both the nutritional status and the functional capacity of the human body. It is beneficial for judging the extent of obesity, providing individualized therapeutic intervention, and evaluating the effectiveness of intervention or treatments [12]. To date, there emerged numerous methods for precise body component analysis, including dual-energy X-ray absorptiometry (DEXA), CT, MRI, bioelectrical impedance analysis (BIA), Whole-body potassium counter, etc. [13]. Though each method has exclusive advantages in body component measuring, the BIA provides spontaneous, convenient, and not expensive estimates in healthy and obese individuals. Therefore, it is more prevalent used in clinics and extensive studies. In this study, we utilized a BIA analyzer (DBA-550; Donghuayuan Medical Co., Ltd, Beijing, China) to evaluate the change and effects of metabolic characteristics and body components by short-term physical training interventions. Using DBA-550, we can compare the effectiveness of the interventions with the other strategies previously reported [14] and provide a more individualized plan for bodyweight control in overweight or obese populations in the future.

## Materials and methods

Ethics statements

The study, including surveys, sampling, and examinations, has obtained ethics approvals from the institute of Ethics Committee of Shanghai Pudong Hospital (Shanghai, China) (WZ-201). The consent was received from study participants before the study, and the guidelines outlined, and procedures followed the Declaration of Helsinki.

Participant's inclusion

Included participants were staff aged 25-50 years old in Shanghai Pudong Hospital, and all participants had been informed. First, we collected the results of annual medical checkups of staff and confirmed the diagnosis of participants with overweight or obesity. Second, we recommended the 10 staff take three-month physical training interventions. All included individuals were without a history of any severe cardiovascular and metabolic diseases, asthma, and disabilities.

Evaluation, starting, and duration of the physical training intervention

All participants received a body metabolic characteristics, body components, and status of job and life stresses evaluation before the intervention. Metabolic characteristics included weight, BMI, circumferences (chest, waist, hip, extremities), blood pressure, ultrasound examinations, blood chemical examinations, and history of physical training, medications, comorbidities, etc.; the body component analysis was conducted on a DBA-550 body composition analyzer; the status of job and life stresses evaluation was achieved in a standard questionnaire (The Physical Activity Readiness Questionnaire, PAR-Q ) on recent job pressure, satisfaction, physical activity, and life stress. After the evaluation, the participants received a three-month intervention containing standard physical training of one hour daily under the guidance of professional training instructors. All training was voluntary and flexible based on the physical status of participants.

Acquisition of data related to metabolic characteristics

We collected data about these participants' metabolic characteristics of weight, BMI, circumferences (chest, waist, hip, extremities), blood pressure, and plasma lipids pre-, one month, and three months of physical training interventions. Before the physical training intervention of participants who completed the project, the general information was shown (Table 1).

**Table 1:** Basic information of participants before physical training intervention Data are Mean ± SD unless otherwise indicated.

Characteristic	Value
Participants (n)	10
Age (years)	35.3±6.929
Female (n)	5
Male (n)	5
Hypertension (n)	5
NAFLD (n)	9
Training history (n)	2
Drug history (n)	3
Family history of obesity (n)	1
Surgery history (n)	2
BMI/ female (Kg/m^2)	31.76±2.495
BMI/ male (Kg/m^2)	31.28±3.459
Systolic pressure (mmHg)	130.50±14.230
Diastolic pressure (mmHg)	89.70±8.084
Chest circumference /female (cm)	105.00±7.969
Chest circumference /male (cm)	108.80±11.606
Waist circumference /female (cm)	100.80±8.228
Waist circumference /male (cm)	106±8.944
Hip circumference /female (cm)	109.6±5.505
Hip circumference /male (cm)	112.80±8.815
Upper arm circumference /female (cm)	32.80±1.789
Upper arm circumference /male (cm)	35.00±3.082
Upper leg circumference/ female (cm)	64.00±3.873
Upper leg circumference/ male (cm)	62.2±4.764
Lower leg circumference/ female (cm)	41.40±1.817
Lower leg circumference/ female (cm)	44.40±4.278

Acquisition of data related to body composition

The data of body components were achieved on a body composition analyzer (DBA-550). All participants received body component analysis before the physical training intervention. We finally collected the body composition data in one month and three months after participating in the training. The body components encompass the weight of the intracellular fluid, extracellular fluid, protein, mineral salt, and body fat, the combinations of which shape the weight of body water, muscle, body weight with or without fat. Other significant indices include skeletal muscle weight, body fat weight, the percentage of fat, waist/hip ratio, organic fat area, segmental muscle analysis, segmental edema indices, etc.

Statistics

Plasma lipids, blood pressure comparisons were performed via two-way analysis of variance (ANOVA) by Sidak multiple comparison tests. The comparisons of BMI were performed via repeat measure one-way ANOVA by Tukey multiple comparison tests. In addition, comparisons of the chest, waist, hip, and limb were conducted via two-way ANOVA by Tukey multiple comparison tests. On the other hand, body components analyses were compared using mixed-effects analysis via Tukey multiple comparison tests. Statistics were performed in SPSS (version 24.0, IBM Corp., Armonk, NY) and Prism (Graphpad, version 8.0; GraphPad Software, Inc, California). For all analyses, significance was assigned at the p < 0.05 level.

## Results

Assessment of plasma lipids, blood pressure, BMI, waist to hip (W/H) ratio, and body circumferences after the intervention

At the point of the third month of physical training intervention, we observed an increase in high-density lipoprotein (HDL), a decrease in low-density lipoprotein (LDL), total cholesterol (TC), and triglyceride (TG) of male participants, and a significant decrease in the level of TG (pre vs. third month: HDL: 1.044±0.168 mmol/l vs. 1.196±0.350 mmol/l; LDL: 3.518±0.867 mmol/l vs. 3.396±0.847 mmol/l; TC: 5.238±0.768 mmol/l vs. 4.860±1.031 mmol/l, p>0.05; TG: 2.432±1.116 mmol/l vs. 1.274±0.547 mmol/l; p<0.05). However, in female participants, there were increases in HDL and TG and decreases in LDL and TC of female participants, but they did not reach a significant level (pre vs 3rd month: HDL: 1.178±0.286 mmol/l vs 1.742±1.090 mmol/l; TG: 1.494±0.433 mmol/l vs 1.526±0.585 mmol/l; LDL: 3.662±1.154 mmol/l vs 3.272±1.853 mmol/l; TC: 5.136±1.173 mmol/l vs 4.638±1.798 mmol/l, p>0.05).

The results of blood pressure measuring seem not significant when compared with it before the intervention, though, in male participants, we observed that decreases in systolic and diastolic blood pressure remained unchanged (pre vs. third month: systolic pressure: 137.4±11.216 mmHg vs. 133.4±14.519 mmHg; diastolic pressure: 87±7.517 mmHg vs. 87±3.391 mmHg, p>0.05); whereas, in female participants, the blood pressure increased ( pre vs. the third month: systolic pressure: 127.8±23.658 mmHg vs. 135.6±14.707 mmHg; diastolic pressure: 85±13.874 mmHg vs. 88.6±8.70 6 mmHg, p>0.05), but did not reach statistical significance.

**Figure 1:**
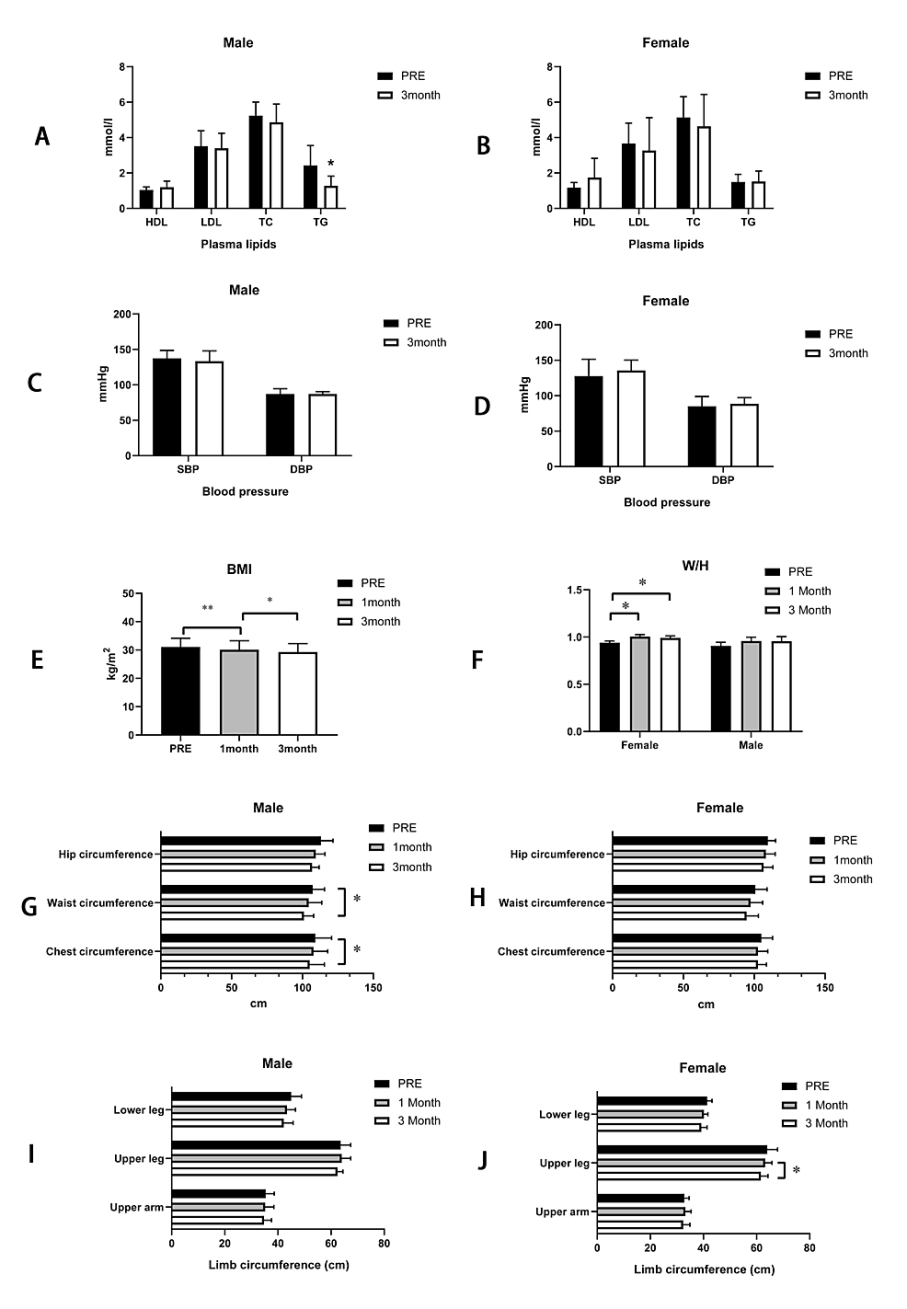
The comparison of plasma lipids, blood pressure, BMI, W/H ratio, body circumference changes in pre- and one and three months after the physical training intervention (A) and (B) show the plasma lipids change after intervention in males and females, respectively; (C) and (D) show blood pressure change after intervention in males and females, respectively; (E) and (F) show the BMI and W/H ratio change after intervention respectively; (G) and (H) show the body trunk circumferences change in males and females after the intervention, respectively; (I) and (J) show the limb circumferences change after the intervention in males and females, respectively. Note: *: p<0.05, **: p<0.01. HDL: high-density lipoprotein; LDL: low-density lipoprotein; TC: total cholesterol; TG: triglyceride; SBP: systolic blood pressure; DBP: diastolic blood pressure; BMI: body mass index; W/H: waist/hip ratio

As for BMI and waist/hip ratio (W/H), we found significant decreases in BMI when compared with the time before physical training interventions in all participants (pre vs 1 month: 31.07±3.085 kg/m^2^ vs 30.09±3.179 kg/m^2^; first month vs third month: 30.09±3.179 kg/m^2^ vs 29.32±2.969 kg/m^2^, p<0.05; Figure 1 (panel E)). In male participants, the W/H decreased not significantly (pre vs 1 month: 0.9060±0.038 vs 0.9575±0.041; first month vs third month: 0.9575±0.041 vs 0.9560±0.048). However, we found significant increases in the W/H ratio of the first month and the third month in female participants when compared with the time before interventions, and W/H decreased in the third month when compared with the first month but did not reach statistical significance (pre vs. one month: 0.94±0.019 vs. 1.005±0.021; one month vs. third month: 1.005±0.021 vs. 0.990±0.022; p<0.05).

The body trunk circumferences showed that in male participants, chest and waist circumferences decreased significantly in the third month compared with those before the intervention, and those changes are not significant when compared with the first month. Also, there was no significance when comparing the first month with those of time before interventions (pre vs 3rd month: chest circumference: 109±11.511 cm vs 105±10.440 cm, waist circumference: 107.2±8.319 cm vs 101±6.892 cm; hip circumference: 113±8.573 cm vs 106.8±4.868 cm, p<0.05; first month vs third month: chest circumference: 107.8±9.985 cm vs 105±10.440 cm, waist circumference: 104.4±9.236 cm vs 101±6.892 cm; hip circumference: 109.4±6.504 cm vs 106.8±4.868 cm; p>0.05). In female participants, the trunk circumference did not decrease significantly (pre vs 3rd month: chest circumference: 105±7.969 cm vs 102.6±6.066 cm, waist circumference: 100.8±8.228 cm vs 94.60±8.385 cm; hip circumference: 109.6±5.505 cm vs 106.6±6.580 cm; first month vs third month: chest circumference: 102.6±6.914 cm vs 102.6±6.066 cm, waist circumference: 97.4±8.562 cm vs 94.60±8.385 cm; hip circumference: 108.2±6.611 cm vs 106.6±6.580 cm; p>0.05).

Similarly, we found the limb circumference did not distinctly change in male participants (pre vs 1st month: upper arm: 35.4±3.209 cm vs 35.2±3.271 cm; upper leg: 63.6±3.782 cm vs 64.0±3.391 cm; lower leg: 45±3.873 cm vs 43.4±3.209 cm; first month vs third month: upper arm: 35.2±3.271 cm vs 34.8±2.775 cm; upper leg: 64.0±3.391 cm vs 62.4±2.074 cm; lower leg: 43.4±3.209 cm vs 42.2±3.493 cm; p>0.05). Also, in female participants, we found only a change in upper leg apparently when comparing those of the first month with the third month (pre vs first month: upper arm: 32.81.789 cm vs 33.2±2.168 cm; upper leg: 64.0±3.873 cm vs 63.2±2.588 m; lower leg: 41.4±1.817 cm vs 40.2±1.483 cm; p>0.05; first month vs third month: upper arm: 33.2±2.168 cm vs 32.4±2.510 cm, p>0.05; upper leg: 63.2±2.588 cm vs 61.6±2.702 cm, p<0.05; lower leg: 40.2±1.483 cm vs 39.2±2.168 cm, p>0.05) (Figure 1).

Body components assessments in pre and first and third months of intervention

The body components analysis (Table 2) showed that in female participants, the mean weight of intracellular fluid (ICF) and extracellular fluid (ECF), which constitute the body water weight decreased after intervention but did not reach statistical levels (ICF: pre: 22.72±1.992 kg; first month: 22.13±3.097 kg; third month: 21.98±3.083 kg; ECF: pre: 13.54±1.182 kg; first month: 12.93±1.727 kg; third month: 12.96±1.649 kg; body water weight: pre: 36.28±3.166 kg; first month: 35.08±4.846 kg; third month: 34.92±4.750kg; p>0.05). In male participants, the mean weight of intracellular fluid (ICF) and extracellular fluid (ECF) decreased after intervention but did not reach a statistical level (ICF: pre: 32.12±4.731 kg; first month: 32.65±5.491 kg; third month: 31.48±5.047 kg; ECF: pre: 19.06±2.798 kg; first month: 18.875±2.978 kg; third month:18.24±2.669 kg; body water weight: pre: 51.2±7.500 kg; first month: 51.475±8.476 kg; third month: 49.74±7.656kg; p>0.05).

**Table 2:** The overview of bodyweight components Note: ICF:intracellular fluid; ECF: extracellular fluid

Components					Normal reference level (Kg)
ICF	Total water	Muscle weight			17.5-21.3
ECF			Bodyweight without fat	Bodyweight	10.7-13.1
Protein					7.7-9.4
Mineral salt					2.74-3.35
Body fat weight					12.3-19.3

The protein component, which plus water, constitutes the weight of muscle was found to fluctuate but not significantly in males (mean protein: pre: 13.90±2.041 kg; first month: 14.10±2.404 kg; third month: 13.60±2.189 kg, p>0.05), whereas, in females, we found that decreased but not significantly (mean protein: pre: 9.820±0.858 kg; first month: 9.575±1.360 kg; third month: 9.480±1.320 kg, p>0.05). Moreover, we found mineral salt, which plus muscle weight, constitutes the body weight without fat, decreased in males (mean mineral salt: pre: 4.850±0.715 kg; first month: 4.740±0.732 kg; third month: 4.594±0.644 kg, p>0.05), and in females, mineral salt fluctuates slightly (mean mineral salt: pre: 3.462±0.302 kg; first month: 3.260±0.427 kg; third month: 3.280±0.401 kg, p>0.05). Both did not reach statistical levels too.

**Figure 2:**
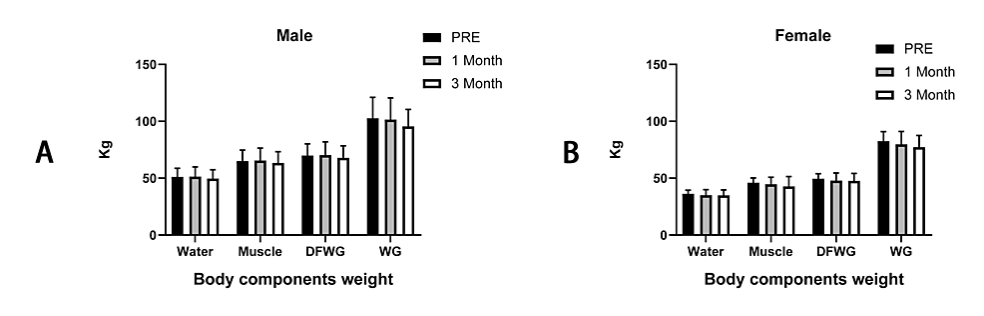
The comparison of body component changes of each participant in male (A) and female participants (B), respectively Note: DFWG: bodyweight without fat; WG: body weight

**Figure 3:**
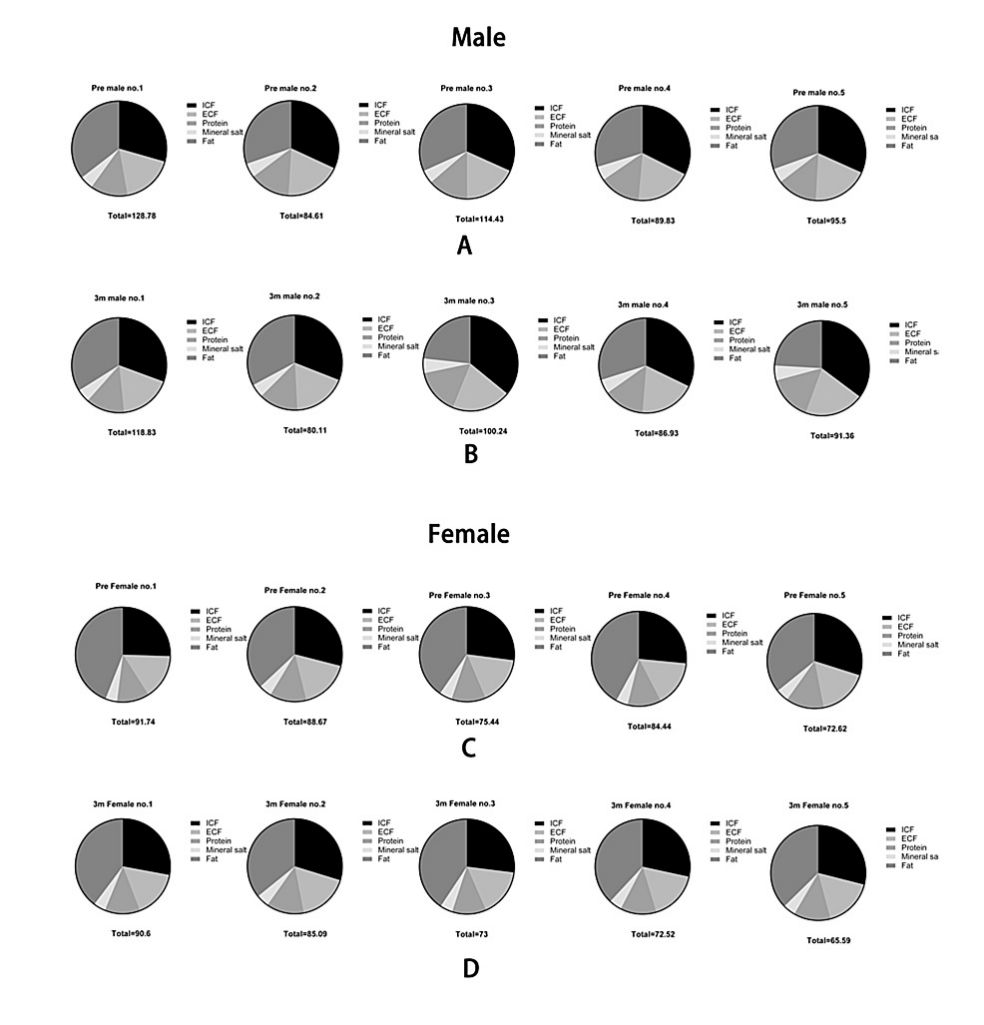
The ratio of body composition change before (A) after intervention (B) in males and females (C and D)

Finally, we found that fat, which is a critical component of body weight, decreased in both males and females but did not reach the statistical level too (mean fat: male: pre: 32.70±8.365 kg; first month: 31.23±9.573 kg; third month: 27.58±7.338 kg; female: pre: 33.04±5.392 kg; first month: 31.88±4.971 kg; third month: 29.66±4.236 kg, p>0.05). The changes in body components and weights in each participant are shown (Figures 2-3).

Muscle, fat, and obese-related indices analyses after the intervention

**Figure 4:**
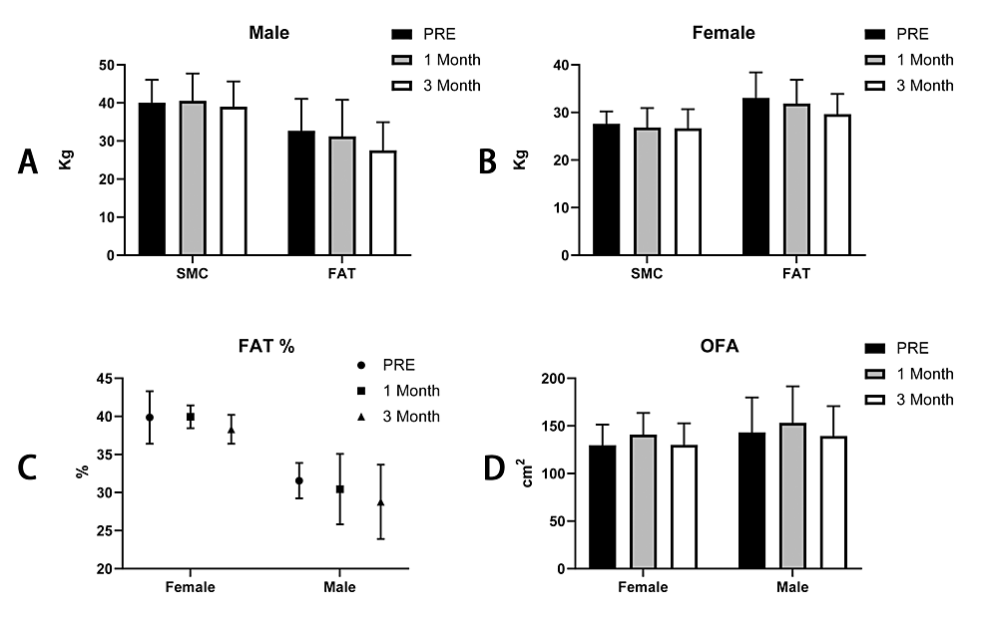
The comparison of skeletal muscle weight and body fat weight in males (A) and females (B) and the percentage of body fat (C) and organic fat area (D) in males and females by the DBA-550 body composition analyzer Note: SMC: the weight of skeletal muscle; FAT: the weight of fat; OFA: organic fat area; FAT%: percentage of body fat

Further, we analyzed the weight of skeletal muscle and body fat weight using muscle-fat analysis. The results showed that in males, the mean skeletal muscle weight fluctuates but not significantly (pre: 40.06±6.019 kg; first month: 40.58±7.155 kg; third month: 39.04±6.595 kg; p>0.05); in female skeletal muscle, the weight decreased but not significantly (pre: 27.62±2.592 kg; first month: 26.85±4.053 kg; third month: 26.68±4.013 kg; p>0.05). Subsequently, we analyzed indices related to obesity, including the percentage of fat and organic fat area. We found that the percentage of fat decreased in males (pre: 31.56±2.317%; first month: 30.45±4.621%; third month: 28.78±4.882%) and fluctuated not significantly but decreased in females (pre: 39.86±3.446%; first month: 39.95±1.507%; third month: 38.30±1.897%). On the other hand, the organic fat area fluctuated not significantly in both males (pre: 143.2±36.752 cm^2^; first month: 153.3±38.255 cm^2^; third month: 139.4±31.263 cm^2^) and in females (pre: 129.8±21.739 cm^2^; first month: 140.9±22.713 cm^2^; third month: 130.1±22.679 cm^2^) (Figure 4).

Segmental muscle analyses after the intervention

**Figure 5:**
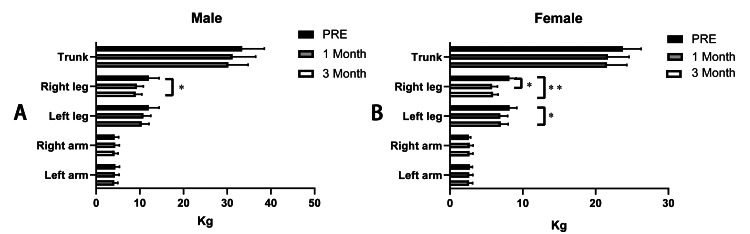
Comparison of segmental skeletal muscle weight defined by left/right arm or leg and trunk in males (A) and females (B) by the DBA-550 body composition analyzer Note: *: p<0.05; **: p<0.01

To determine the effects of the short-term intervention on the quality of muscles, we analyzed the segmental skeletal muscle weight defined by the left/right arm, left/right leg, and trunk. We found that in males, only the muscle weight of the right leg significantly decreased (pre: 12.07±2.332 kg; third month: 9.072±1.391 kg, p<0.05). However, we found that in female participants, the muscle weight of both the left and right legs decreased significantly when comparing those of the third month with pre-intervention (left leg: pre: 8.190±0.988 kg; third month: 6.976±1.034 kg, p<0.05; right leg: pre: 8.154±0.939 kg; third month: 5.922±0.691 kg, p<0.05), and when comparing the first-month muscle weight of the right leg with pre-intervention, it decreased significantly as well (right leg: pre: 8.154±0.939 kg; first month: 5.788±0.741 kg, p<0.05) (Figure 5).

Segmental edema indices analyses after the intervention

In order to assist in judging the body water distribution and skeletal muscle content, we assessed the segmental edema indices, which include extracellular fluid/total body fluid (ECF/TBF) and extracellular water/total body water (ECW/TBW). We found that except the trunk and total ECF/TBF, there were significant increases when either comparing ECF/TBF of the first month or third month with those of the pre-intervention in males (left arm: pre: 0.3160±0.005; first month: 0.3275±0.005; third month: 0.3280±0.004; p<0.05; right arm: pre: 0.3160±0.005; first month: 0.3275±0.005; third month: 0.3280±0.004; p<0.05; left leg: pre: 0.3180±0.008; first month: 0.3325±0.010; third month: 0.3340±0.009; p<0.05; right leg: pre: 0.3140±0.009; first month: 0.3325±0.010; third month: 0.3340±0.009; p<0.05; trunk: pre: 0.3280±0.008; first month: 0.3375±0.005; third month: 0.3380±0.004; p>0.05; total: pre: 0.3240±0.005; first month: 0.3150±0.006; third month: 0.3180±0.004; p>0.05;), but all of ECF/TBF were within the normal range (0.29-0.37); in females, we also found that only total ECF/TBF was not significant; there were significant increases when either comparing ECF/TBF of the first month or third month with those of pre-intervention in females (left arm: pre: 0.3160±0.005; first month: 0.330; third month: 0.330; p<0.05; right arm: pre: 0.3180±0.004; first month: 0.330; third month: 0.3300; p<0.05; left leg: pre: 0.3180±0.008; 1st month: 0.340±0.008; third month: 0.3400±0.007; p<0.05; right leg: pre: 0.3160±0.009; first month: 0.340±0.008; third month: 0.3420±0.008; p<0.05; trunk: pre: 0.332±0.004; first month: 0.343±0.005; third month: 0.342±0.004; p<0.05; total: pre: 0.322±0.008; first month: 0.32; third month: 0.322±0.004; p>0.05;), but all of ECF/TBF were within the normal range (0.29-0.37).

**Figure 6:**
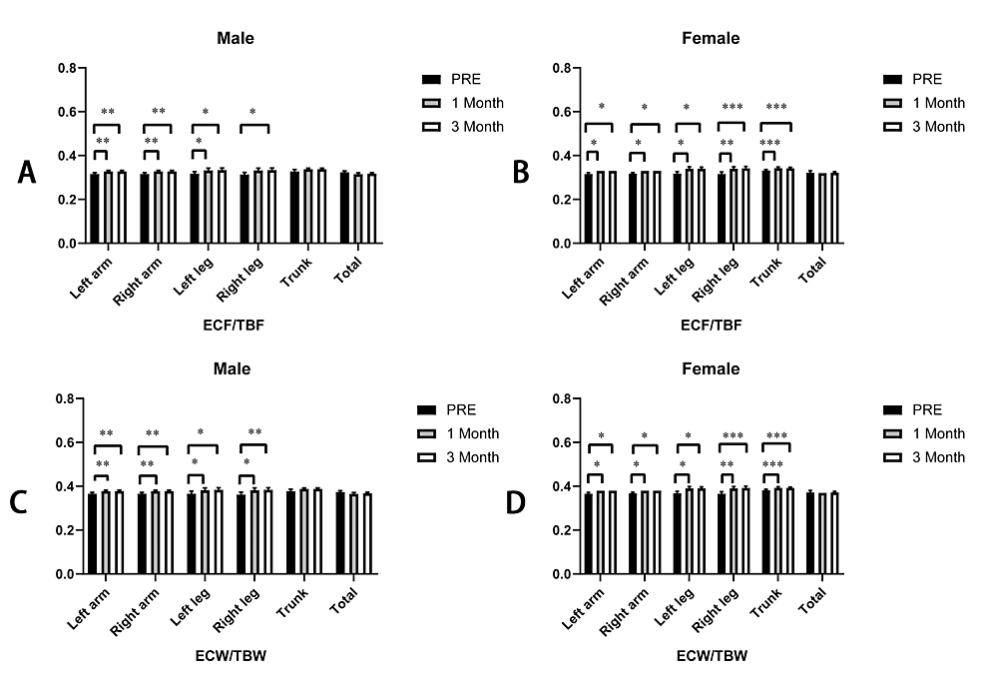
Comparison of ECF/TBF in males (A) and females (B); ECW/TBW in males (C) and females (D) Note: *: p<0.05; **: p<0.01; ***: p<0.001. ECF/TBF: extracellular fluid/ total body fluid; ECW/TBW: extracellular water/ total body water

As for ECW/TBW, we observed that except the trunk and total ECW/TBW, there were significant increases when either comparing ECW/TBW of the first month or third month with those of pre-intervention in males (left arm: pre: 0.366±0.005; first month: 0.378±0.005; third month: 0.378±0.004; p<0.05; right arm: pre: 0.366±0.005; first month: 0.378±0.005; third month: 0.378±0.004; p<0.05; left leg: pre: 0.366±0.011; first month: 0.383±0.010; third month: 0.384±0.009; p<0.05; right leg: pre: 0.362±0.011; first month: 0.383±0.010; third month: 0.384±0.009; p<0.05; trunk: pre: 0.378±0.008; first month: 0.388±0.005; third month: 0.388±0.004; p>0.05; total: pre: 0.374±0.005; first month: 0.365±0.006; third month: 0.386±0.004; p>0.05;), but all of ECW/TBW were within the normal range (0.34-0.42); in females, we also found that only total ECW/TBW was not significant; there were significant increases when either comparing ECW/TBW of the first month or third month with those of pre-intervention in females (left arm: pre: 0.366±0.005; first month: 0.38; third month: 0.38; p<0.05; right arm: pre: 0.368±0.004; first month: 0.38; third month: 0.38; p<0.05; left leg: pre: 0.368±0.008; first month: 0.390±0.008; third month: 0.390±0.007; p<0.05; right leg: pre: 0.366±0.009; first month: 0.390±0.008; third month: 0.392±0.008; p<0.05; trunk: pre: 0.382±0.004; first month: 0.393±0.005; third month: 0.392±0.004; p<0.05; total: pre 0.372±0.008; first month: 0.370; third month: 0.372±0.004; p>0.05;), but all of ECF/TBF were within the normal range (0.34-0.42) (Figure 6).

## Discussion

Diet and exercise are two essential interventions in the management of obesity [15]. Obesity is often accompanied by metabolic syndrome, and if it is not well controlled, may lead to severe complications [4] or sudden death [16]. In the early phase, diet and exercise display powerful effects and can even reverse a few complications [17-18]. However, appropriate calorie restrictions and suitable loads of energy expenditure can benefit organs and prevent a few weight-loss-induced complications such as non-alcoholic fatty liver diseases (NAFLD). Therefore, we utilized a body components analyzer combined with blood sampling or ultrasound check to reveal the body components change and effectiveness of three months of physical training interventions. We observed many improvements in metabolic characteristics, such as plasma lipids, BMI, trunk circumferences, and body components, including organ fat area and percentage of fat, indicating the positive effects of the intervention on the overall metabolism in staff. Through this study, we desire to establish a more practical and effective sports strategy for obesity resistance in our hospital staff and may add our study results to exercise plan selection in obesity management.

First, we found positive intervention effects on the significant metabolic characteristics represented by plasma lipids, BMI, and chest circumference. Among the other indices, BMI is the most critical general obesity index; we found significant reductions in all participants compared with the time before the intervention. Compared with subsequent body component analyses, especially reduction of fat weight, we found this intervention may primarily lead to fast weight reduction, thus virtually reflecting BMI reduction. Among many indices, through blood sampling, we observed a meaningful plasma lipids improvement after the intervention, though the change in females did not reach a statistical level, suggesting the potential of intervention alone to improve lipid metabolism. Blood pressure seems to only significantly decrease in male participants. In older populations, we often found hypertension, differences in hormonal environment, and the duration and pattern of exercise [19] between males and females. Thus, we may only observe the distinct protective role of exercise in systolic blood pressure in male participants. Although only the chest and waist circumference change reached a statistical level in male participants, we still found the most circumference declined as the result of the intervention. This phenomenon may be attributed to the different physical training patterns between males and females and the enhanced metabolic rate in male participants. Limb circumference changed not significantly, suggesting the intervention did not cause malnutrition since limb circumference reflects the nutrition status of participants [20].

After then, we utilized a DBA-550 body composition analyzer to detect and analyze the body component change. We first found that ICF and ECF, which constitute the body water weight, decreased. The average body water accounted for 50%-60% of the body weight. The ratio of ICF to ECF is generally 3:2. In conditions like kidney disease, hypertension, cardiovascular diseases, general or local swelling, malnutrition, water homeostasis is always imbalanced. The ratio of ICF to ECF in this study remained within the normal range, suggesting the weight loss could relieve increased plasma tonicity but did not induce water imbalance [21]. The protein content primarily reflects the quality of the skeletal muscle after intervention [22]; we may find that, unlike the male participants, in females, the protein loss decreased but did not reach a statistical level; one reason may be that the training pattern varies between male and female, which kept skeletal muscle stable in male participants but caused the continued muscle contents losses in female participants.

Conversely, the mineral salt, which is the critical component for skeletal [23], decreased in males but fluctuated in females, which may also be attributed to the training pattern. Therefore, the supplement of nutrients after training would be essential. However, despite differential training patterns, both resulted in fat mass loss, suggesting the effectiveness of the intervention. Further, we analyzed the obese-related indices, including organic fat area (OFA) and fat percentage. OFA refers to visceral fat in the abdominal cavity, when the section area is more than 100cm^2^, it is considered abdominal obesity and is highly prone to complications like cardiovascular diseases [24]. The visceral fat always uneasily disappears [25-26] consistently; we also found that the fat percentage was lower in males as previously reported [27] and decreased in all genders, suggesting long-term exercise may be needed to improve the fat of visceral organs. In the next step, we analyzed the effects of the intervention on the quality of the muscles. We only found a minor decrease in the muscle of limbs, suggesting the intervention did not significantly influence the quality of overall muscles. Finally, the body components analyzer showed that either the ECF/TBF or the ECW/TBW of all participants were within the normal range, suggesting the intervention did not induce local or whole-body circulation abnormality [28-29].

Limitations

This study only summarized 10 participants due to the long periods of the training that make merely a few participants complete this mission. In addition, we did not follow up to six months and one year to observe the final significant effects of body components changes. Larger contents of the sample and follow-up period will be needed in the future. However, the body composition analyzer is a useful and convenient tool to monitor the systemic metabolic change after interventions.

## Conclusions

In conclusion, we found that the three-month, short-term physical training intervention caused significant loss of BMI and plasma lipids, caused decreases in the trunk circumference, and led to body composition alterations, especially in the percentage of body fat, without inducing malnutrition or circulation abnormalities. This study suggests short-term physical interventions may be a helpful strategy for bodyweight management, and it provides merits to metabolism although we should supplement some mineral salts after training to prevent bone content loss. The body composition analyzer is a valuable and accurate tool to evaluate the effectiveness of the intervention, can assist in modifying more individualized treatment plans for overweight and obesity. Our present study also found that in the tertiary hospital, bodyweight management in staff is essential. Due to the high-intensity workload, many staff, especially younger workers, may neglect the implication of exercise and diet control. Thus, we hope that our study may provide scientific insights on the strategy to manage overweight or obesity and evoke corresponding measures to effectively alleviate the work stress and stop the prevalence of overweight and obesity among young workers of Chinese hospitals.
